# Editorial: Advancing surgical outcomes for retroperitoneal tumors

**DOI:** 10.3389/fsurg.2026.1996344

**Published:** 2026-09-24

**Authors:** Aobo Zhuang, Bin Zhou, Mengmeng Xiao

**Affiliations:** 1 Cancer Research Center, School of Medicine, Xiamen University, Xiamen, China; 2 Department of Hepatobiliary and Pancreatic Surgery, The Affiliated Hospital of Qingdao University, Qingdao, Shandong, China; 3 Department of General Surgery, Peking University People’s Hospital, Beijing, China

**Keywords:** multidisciplinary care, perioperative management, precision surgery, rare tumors, retroperitoneal tumors, surgical oncology

Retroperitoneal tumors sit at the intersection of anatomical difficulty and biological diversity. Their deep location can delay recognition, while their relationships with major vessels, viscera, nerves, and the musculoskeletal system make treatment decisions inseparable from questions of function and perioperative risk. This Research Topic brings together 21 articles: six original studies, 14 case reports, and one review. The Research Topic spans primary retroperitoneal and pelvic neoplasms, related abdominal tumors, and technically instructive operations outside the retroperitoneum. Although heterogeneous, these contributions converge on a shared clinical pathway: improve diagnostic resolution, plan around patient-specific anatomy, scale treatment to tumor biology, and coordinate surgery with longitudinal multidisciplinary care.

## From diagnostic uncertainty to actionable anatomy

The first recurring message is that operative quality begins before the incision. Zheng et al. developed an ultrasonography-based model and simplified score to distinguish retroperitoneal ganglioneuroma from schwannoma in 74 patients. Optimism-corrected areas under the curve were 0.930 and 0.917, respectively, but the authors appropriately called for external validation and recalibration before routine use. In a cohort of 28 adults with primary retroperitoneal lymphangioma, Li et al. described characteristic multiloculated cystic imaging features, complete resection in all patients, and no recurrence or disease-specific death during a median follow-up of 76 months. Yang et al. similarly showed how pathology can resolve the marked clinical heterogeneity of pelvic solitary fibrous tumors: three patients underwent individualized open, robot-assisted, or laparoscopic R0 resection, with nuclear STAT6 supporting diagnosis. These series are small, but they illustrate how imaging, operative anatomy, and pathology should be interpreted as one continuous diagnostic process.

Moving beyond pairwise discrimination, Feng et al. developed REMIND, an end-to-end deep learning model for contrast-enhanced computed tomography, using data from 12 Chinese centers. The model was trained in 606 patients, externally validated in 736, and prospectively evaluated in 188; it combined seven-class diagnosis with tumor segmentation and, in a reader study, achieved a top-1 accuracy comparable to senior radiologists while improving the performance of less-experienced readers (1). This work suggests that artificial intelligence may become a useful decision-support layer for rare, heterogeneous retroperitoneal neoplasms. Before such systems influence biopsy or operative strategy, however, their calibration, failure modes, and transportability should be tested prospectively in other populations and clinical workflows.

Complex anatomy also benefits from explicit reconstruction. In a case of ganglioneuroma involving an ectopic inferior vena cava, Xu et al. used three-dimensional reconstruction to plan robot-assisted resection. Such tools should not be treated as decorative imaging. Their value lies in defining displacement, encasement, collateral pathways, and the likely need for vascular control before committing to an approach.

Several reports show why apparent natural history or an unusual syndrome must not replace tissue diagnosis. Spontaneous radiological regression did not exclude malignancy in a Bosniak IV cystic renal lesion, which proved to be clear cell renal cell carcinoma after partial nephrectomy. In an adolescent with a large renal mass and a pathogenic TSC2 variant, malignant perivascular epithelioid cell tumor was distinguished from angiomyolipoma only after histopathological and molecular assessment. Adult pelvic fibrosarcoma presenting with bowel obstruction required an exclusionary pathological workup, while refractory mucocutaneous disease in a patient with retroperitoneal dedifferentiated liposarcoma was ultimately recognized as paraneoplastic pemphigus.

The limits of inference from isolated PEComa cases are clarified by the TARPSWG study of Mor et al., which included 77 patients with primary extra-visceral retroperitoneal or abdominopelvic PEComas from 13 centers. Among 59 patients treated with curative intent, 24 recurred; outcomes differed by histological subtype, with PEComa not otherwise specified showing the highest recurrence and mortality, sclerosing PEComa an intermediate course, and angiomyolipoma predominantly indolent behavior (2). This pattern supports expert pathology review, biopsy-informed classification, and subtype-adapted surveillance rather than risk assessment based on tumor size alone. Together with the cases in this collection, it argues for early multidisciplinary review whenever symptoms, imaging, pathology, and presumed biology do not align.

## Tailored surgery: radical where necessary, restrained where possible

The second message is not maximal surgery, but appropriately scaled surgery. For dedifferentiated liposarcoma with true colonic infiltration, tumor resection with right hemicolectomy addressed contiguous disease and achieved short-term control. For advanced low-grade serous ovarian carcinoma extending to the iliac vessels and paraspinal region, coordinated gynecologic, orthopedic, plastic, and gastrointestinal surgery enabled R0 resection, femoral vein repair, and flap reconstruction. The proposed “Four Surgical Limits” framework - anatomical, oncological, functional, and interdisciplinary - offers a useful language for determining when apparent technical limits can be crossed safely.

The same principle applies to severe benign disease. A giant uterine leiomyoma complicated by pleural and ascitic effusions, venous thromboembolism, hernia, and pelvic organ prolapse was treated through staged stabilization followed by tumor resection. Conversely, intravenous leiomyomatosis extending through the inferior vena cava into the right atrium demonstrated that even successful *en bloc* removal does not end perioperative risk; postoperative pulmonary embolism demanded rapid recognition and treatment. These reports emphasize that resectability is not simply an anatomical label. It is a team judgment that includes physiological reserve, thrombotic risk, reconstructive options, and rescue capacity.

Quantifying that risk may make perioperative planning more transparent. In 319 patients undergoing resection of primary retroperitoneal sarcoma, Zhuang et al. developed an internally validated morbidity nomogram incorporating age, tumor burden and location, operative time, the number and weighted score of resected organs, estimated blood loss, and packed red-cell transfusion. The model had a concordance index of 0.795 (95% confidence interval 0.746–0.844) (3). It offers a structured basis for risk stratification, counselling, and postoperative vigilance, but several inputs become known only during surgery. A clinically mature pathway will therefore need separately validated preoperative and dynamic intraoperative tools, with external calibration before they are used to alter treatment selection.

Treatment intensity should also reflect natural history. In a retrospective series of 76 patients with desmoid tumors, one-year progression was similar after surgery and active surveillance, while outcomes after R0 resection were favorable over longer follow-up. Because allocation was nonrandomized, these findings do not establish equivalence. They do, however, support contemporary risk-adapted selection: observation is reasonable for some patients, while surgery remains valuable when symptoms, progression, morbidity, or achievable local control justify intervention.

Several technically diverse contributions extend this patient-tailored philosophy. Embryonic natural orifice transluminal endoscopic surgery achieved mucosa-preserving full-thickness muscular resection in 12 beagles, providing preclinical feasibility rather than a mandate for human adoption. Arthroscopy-assisted curettage and bone grafting preserved hip function in a proximal femoral giant cell tumor. Laparoscopic appendectomy in congenital visceral heterotaxy with severe adhesions showed the importance of preoperative mapping and intraoperative adaptability, while recurrent intussusception caused by ileal angiolipoma required definitive bowel resection after transient endoscopic reduction. These articles are not all about primary retroperitoneal tumors, but their transferable lesson is directly relevant: minimally invasive access, organ preservation, and conversion thresholds should be chosen around anatomy and disease behavior, not around a preferred platform.

## Surgery as one component of longitudinal care

The third message is that an operation must be judged by its place in the entire disease course. The phase 3 STRASS trial provides an essential benchmark for multimodality treatment. Among 266 patients with primary retroperitoneal sarcoma, preoperative radiotherapy plus surgery did not improve abdominal recurrence-free survival compared with surgery alone (median 4.5 vs. 5.0 years; hazard ratio 1.01, 95% confidence interval 0.71–1.44), while serious adverse events were more frequent in the combined-treatment group (24% vs. 10%) (4). The negative primary result argues against routine preoperative radiotherapy across all histologies; any histology-specific rationale should be discussed as selective and multidisciplinary rather than as a reversal of that result.

Cytoreductive surgery for treatment-refractory extramedullary plasmacytoma achieved local wound control without reversing systemic progression, underscoring the need to define palliative goals before intervention. In malignant peritoneal mesothelioma treated with cytoreductive surgery and hyperthermic intraperitoneal chemotherapy, a retrospective comparison suggested improved survival with postoperative bevacizumab plus pemetrexed/platinum chemotherapy; the nonrandomized design makes prospective confirmation essential. For heavily pretreated myxoid pleomorphic liposarcoma, eribulin, sintilimab, and radiotherapy produced a partial response lasting 12 months in a single patient. This is a clinically meaningful signal in an ultra-rare disease, but not yet a treatment standard.

Jia et al. looked further ahead, proposing that transcriptomic subtyping, surgical risk stratification, and artificial intelligence-guided drug repurposing could be integrated in retroperitoneal leiomyosarcoma. The framework is conceptually attractive because anatomic complexity and molecular heterogeneity are currently assessed in parallel rather than jointly. Its translation will require reproducible subtype definitions, prospective biospecimen collection, pharmacological validation, and trials designed around clinically plausible treatment windows.

## A research agenda beyond the case report

The collection's strength is its capture of rare presentations that large trials may never describe. Its limitation is the same: 14 of 21 contributions are case reports, and several original studies are retrospective, single-center, or preclinical. Short follow-up without recurrence, successful R0 resection, and an exceptional drug response are valuable observations, but none should be generalized beyond their evidentiary level.

The next phase of progress should therefore convert accumulated experience into comparable data. Multicenter registries should use common definitions for histology, margin status, organ resection, vascular involvement, complications, patient-reported outcomes, recurrence pattern, and long-term function. Diagnostic and risk models require geographic and institutional external validation. Three-dimensional imaging and standardized radiological descriptors should be evaluated for their effect on decisions and outcomes, not only for technical feasibility. Molecular profiling should be embedded in prospective cohorts and therapeutic trials, particularly for recurrent or unresectable sarcoma. Negative findings, aborted procedures, conversions, and serious complications deserve the same reporting discipline as technical success.

Advancing outcomes for retroperitoneal tumors will not come from a single device, operative approach, or drug. It will come from linking diagnostic precision to anatomical planning, matching the extent of surgery to biology and function, and measuring success over the patient's full trajectory. The articles in this Research Topic provide instructive building blocks for that shift and, equally importantly, clarify the evidence still needed to make it durable.
